# Antibacterial Mechanism of Action of Two Types of Honey against *Escherichia coli* through Interfering with Bacterial Membrane Permeability, Inhibiting Proteins, and Inducing Bacterial DNA Damage

**DOI:** 10.3390/antibiotics11091182

**Published:** 2022-08-31

**Authors:** Asma Mohammed Al-Sayaghi, Abdelkodose Mohammed Al-Kabsi, Maisa Siddiq Abduh, Sultan Ayesh Mohammed Saghir, Mohammed Abdullah Alshawsh

**Affiliations:** 1 Faculty of Medicine, University of Cyberjaya, Persiaran Bestari, Cyberjaya 63000, Malaysia; 2 Department of Medical Laboratory Sciences, Faculty of Applied Medical Sciences, King Abdulaziz University, Jeddah 21589, Saudi Arabia; 3 Center of Excellence in Genomic Medicine Research, King Abdulaziz University, Jeddah 22252, Saudi Arabia; 4 Department of Medical Analysis, Princess Aisha Bint Al-Hussein College of Nursing and Medical Sciences, Al-Hussein Bin Talal University, Ma’an 71111, Jordan; 5 Department of Pharmacology, Faculty of Medicine, Universiti Malaya, Kuala Lumpur 50603, Malaysia

**Keywords:** Yemeni Sidr honey, Manuka honey, antibacterial mechanism of action, *Escherichia coli*

## Abstract

Honey is a sweet natural food produced by bees from flower nectar or some part of plant secretions that exhibit antimicrobial activity against many microorganisms. It has been used as traditional therapy for skin infections. Antibiotics play an essential role in managing wound infection; however, some pathogenic bacteria have begun to possess resistance against them, which may cause chronic infections and severe adverse effects. This study investigates the antibacterial activities and mechanism of action of Yemeni Sidr honey (SH) and Manuka honey (MH) against *Escherichia coli*. The inhibitory effects of SH and MH using the disk diffusion method on bacterial growth were remarkable at 700 mg/disk. The minimum inhibitory concentration (MIC) and minimum bactericidal concentration (MBC) were similar for both kinds of honey. However, MH showed a better bactericidal effect (30%) than SH (50%). The antimicrobial mechanism of action showed that SH substantially impacted the bacterial membrane’s permeability and increased the potassium and protein leakage rate. On the contrary, MH demonstrated remarkable inhibition of bacterial protein synthesis, while both kinds of honey caused bacterial DNA damage. These data reveal that SH and MH could be used as a remedy for skin infections and might be further developed as a promising dressing for bacterial wound infections.

## 1. Introduction

Honey is a nutritional product produced by bee species from various medicinal plants; their botanical properties are transmitted through honey as a vehicle [1]. The antimicrobial efficacy of honey is complicated and still has incomplete recognition. However, it has been confirmed that several substances presented in honey play an essential role in antimicrobial potencies related to their physical (acidity and osmolarity) as well as chemical properties (bioactive compounds) [2]. Hydrogen peroxide (H_2_O_2_) is one of the main components in most types of honey responsible for antibacterial activities, which has antiseptic properties and is a strong oxidizing agent against microorganisms [3]. Besides H_2_O_2_, some of the distinguished bioactive constituents found in honey also have an active role in antibacterial effectiveness, which is called non-peroxide honey; these are bioactive compounds such as dicarbonyl methylglyoxal (MGO), polyphenols and bee peptides (bee defensin-1) [4,5]. Non-peroxide honey (certain Manuka honey) exhibits antibacterial properties even when H_2_O_2_ production is blocked [6]. Polyphenols are secondary metabolites derived from plants; these biological compounds are transmitted from nectar to honey and are crucial for the health-promoting characteristics of honey [7]. Polyphenols are variable chemicals distinguished by their phenolic structures and include flavonoids and phenolic acids. Honey contains a high concentration of phenolic substances, which may contribute to its antibacterial effect [7].

Most bacteria grow best at neutral pH levels ranging from 6.5 to 7.5; honey’s acidity ranges from 3.5 to 5.5, a distinguishing feature considered low enough to inhibit much bacterial growth [8]. Certain essential organic acids, particularly gluconic acid, are powerful antibacterial agents produced by glucose oxidation by an endogenous glucose oxidase enzyme [5,9]. Alongside its acidic properties, its high osmolarity (high sugar contents), causing high osmotic pressure, kills/inhibits plenty of microorganisms [5]. Last and not least, the diversity of the antibacterial activity of honey depends on its botanical origin, geographical region, types, and climate conditions.

Wound infections are among the most common diseases in developing countries and usually are caused by unsanitary environments [10]. Wound infection is a major complication of injury, particularly burns, and contributes to 50% to 75% of hospital mortality [11]. The pathogenic bacteria play a principal role in delayed wound healing, slowing the healing processes in the injured area. Even though antibiotics play an essential role in managing wound infection, some pathogenic bacteria become resistant, which may cause chronic infections and severe adverse effects. In many cases of poor healing, the most common bacteria found in the wound area is *Escherichia coli* [12]. Researchers have invested a massive effort to reveal the effectiveness of Manuka honey (MH) from New Zealand in several application fields, such as wound healing, antimicrobial activities [13], antioxidant properties, antiviral, anticancer, and other pharmacological properties [14,15,16,17]. In addition, several studies have been carried out to explore the components and characterization of MH. On the other hand, Yemeni Sidr honey (SH) from Yemen is not as popular as MH, with limited scientific reports and insufficient investigation of its antimicrobial mechanism of action because this type of honey is unknown worldwide and considered local honey. Hence, this study aimed to compare the antimicrobial activities and mechanism of action of Sidr and Manuka honey.

## 2. Results

### 2.1. Antibacterial Susceptibility Assay

**Figure 1:**
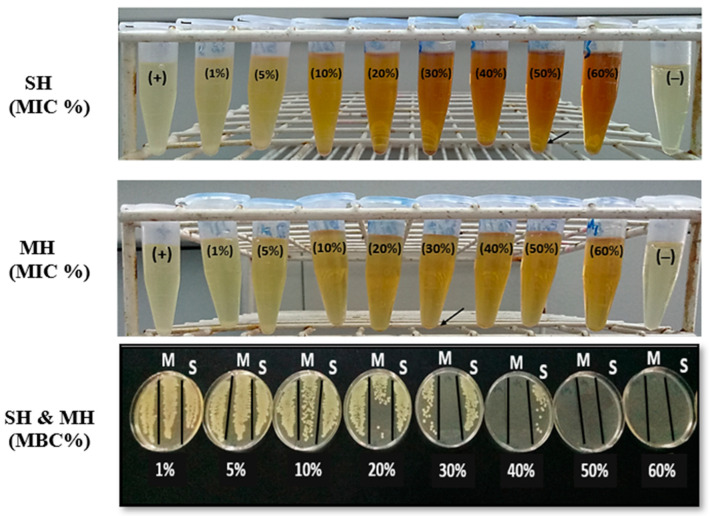
Photos show the minimum inhibitory concentration (MIC) and minimum bactericidal concentration (MBC) of Sidr honey (SH) and Manuka honey (MH); (+): positive control (bacteria with broth medium); (–): negative control (only broth medium).

Both SH and MH inhibited the bacterial growth at 700 mg/disc, with an inhibition zone of 16.33 ± 0.33 mm (SH) and 16.66 ± 1.20 mm (MH) compared with the positive control at 16.5 ± 1.04 mm (gentamicin 10 µg/disc). The minimum inhibitory concentration (MIC) of SH and MH was 50% and 30%, respectively, whereas the minimum bactericidal concentration (MBC) was the same as the MIC for both honey types, as shown in Figure 1.

### 2.2. Effect of Honey on the Potassium (K^+^) Ion Leakage from the Cell Membrane of Escherichia coli

**Figure 2:**
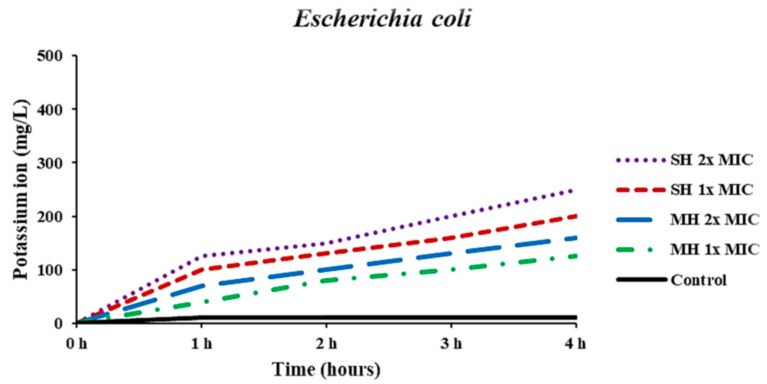
Effect of Sidr honey (SH) and Manuka honey (MH) at 1x MIC and 2x MIC and control on leakage of potassium ions from *Escherichia coli*.

The permeability of the bacterial cell membrane was evaluated based on the leakage of free potassium (K+) ions from *E. coli* treated with honey samples. The negative control (bacterial suspension) showed relatively no changes in potassium ion concentrations during the 4 h of incubation compared to baseline (0 h); however, *E. coli* treated with SH and MH at 1x and 2x MIC showed that all concentrations noticeably increased the potassium leakage from the bacterial cell membrane in a time-dependent manner. However, SH in both concentrations exhibited a higher rate of potassium leakage rate than MH (Figure 2).

### 2.3. Effect of Honey on the Protein Leakage from the Cell Membrane of Escherichia coli

**Figure 3:**
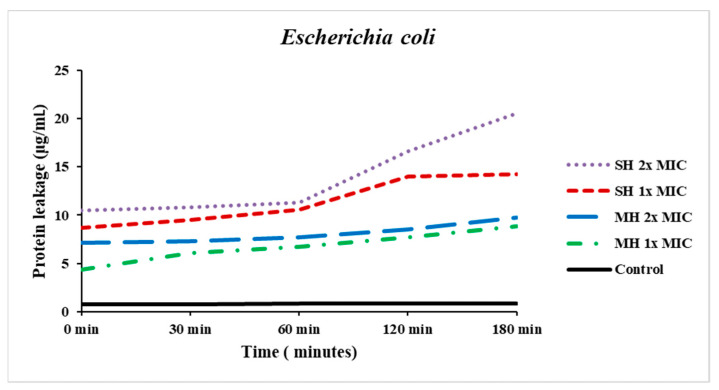
Effect of Sidr honey (SH) and Manuka honey (MH) at 1x MIC and 2x MIC and control on leakage from *Escherichia coli*.

The quantity of protein leakage increased in a time-dependent manner from the baseline (0 min) until 180 min. SH at 2x MIC showed higher protein leakage at 120 min (16.618 ± 0.008 µg/mL) and reached 20.513 ± 0.007 µg/mL at 180 min compared to control, whereas, at 1x MIC, the protein leakage at 120 min was 13.996 ± 0.008 µg/mL and then increased slightly to 14.221 ± 0.012 µg/mL at 180 min. On the other hand, MH at 1x MIC and 2x MIC caused a gradual increase in protein leakage from the cell membrane (Figure 3).

### 2.4. Inhibitory Effect of Honey on the Protein Synthesis of Escherichia coli

**Figure 4:**
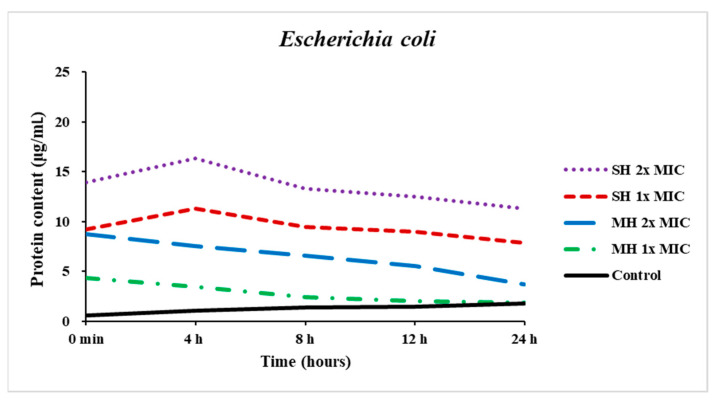
Effect of Sidr honey (SH) and Manuka honey (MH) at 1x MIC and 2x MIC and control on the bacterial protein content of *Escherichia coli*.

The effect of SH and MH on the intracellular protein of *Escherichia coli* is shown in Figure 4. The protein content of the control (untreated bacterial suspension) increased slightly at 24 h compared to the baseline. However, the protein content of bacteria treated with both concentrations of SH slightly increased at the first 4 h, then decreased between 8 h and 24 h. On the other hand, the bacterial protein content of *E. coli* treated with MH at 1x and 2x MIC showed a gradual reduction from the first hours to the end of the experiment.

### 2.5. Bacterial DNA Damage of Escherichia coli by Honey

**Figure 5:**
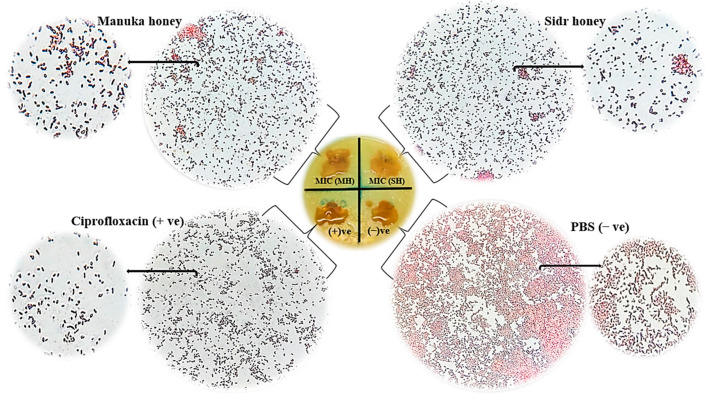
Effect of Sidr honey (SH) and Manuka honey (MH), (+ve): positive control (10 μg/mL Ciprofloxacin), and (−ve): negative control (PBS) on DNA damage of *Escherichia coli*.

The damage to the bacterial DNA genome caused by honey was demonstrated by using a DNA-damaging reagent (6-Bromo-2-3 naphthyl B-d-galactopyranoside), which reacts with the enzyme β-galactosidase activity (LacZ gene) to yield an intensive deep purple color (positive result) around the test compound. This method was applied to *E. coli,* as indicated in Figure 5. The results of this experiment revealed that no color spots appeared on the surface of the culture agar inoculated with bacteria after being treated with honey samples, positive control (ciprofloxacin 10 µg/mL) and negative control (PBS). However, under the microscope, a dark purple color was observed, indicating the hydrolysis of chromogenic reagent by the β-galactosidase enzyme. In addition, shrinkage or changes in the shape of bacteria were observed in the positive control compared to the negative control. Both SH and MH revealed markedly affected bacterial DNA (dark purple color indicates the destruction of DNA, Figure 5). Both honey samples can destroy bacterial nucleic acid similarly, as seen in the positive control.

## 3. Discussion

Honey has antibacterial efficacy against abundant bacterial strains in various environments. The bioactive constituents of honey exhibited a variety of antimicrobial activities against microbes. The differences in the spectrum of antimicrobial effects of honey were perhaps related to the chemical composition of floral nectar and the pasture where the bees foraged [18]. Many studies have reported that MH is the best known among other types of honey and is sold with standardized levels of antibacterial activity. It has been reported that MH has inhibitory effects against approximately 60 species of bacterial strains, including antibiotic-resistant Gram-positive and -negative bacteria [19]. The findings of our study showed that SH and MH (700 mg/disc) could inhibit the bacterial growth of *E. coli*. The inhibition zone diameters of SH and MH were 16.33 ± 0.33 mm and 16.66 ± 1.20 mm, respectively, similar to the positive control (gentamicin 16.5 ± 0.40 mm). Our findings agree with a study conducted by Saeed and Jayashankar [20], who tested SH against clinically isolated *E. coli* at a concentration of 80% (1300 mg/disc). On the contrary, SH in that study showed some antibacterial activity against the other clinical isolated bacterial strains, including MSSA (15 ± 0.08 mm), *Pseudomonas aeruginosa* (14 ± 0.15 mm) and methicillin-resistant *Staphylococcus aureus* (MRSA) (16 ± 0.45 mm). Compared with the Saudi Sidr honey, a study conducted by Graham et al. [3] used 80% *w/v* of the honey solution against *E. coli,* and the inhibition zone diameter was 14.00 ± 1.0 mm. Other bacterial strains such as *Proteus mirabilis*, *Shigella flexneri*, and *Staphylococcus epidermidis* had inhibition zones ranging from 25.33 mm to 37.33 mm. MH showed antibacterial efficacy against tested clinical isolates of *Streptococcus* group D, *Staphylococcus aureus*, *Escherichia coli*, *Salmonella typhymurium* and *Salmonella enterica* [21]. The minimum inhibitory and bactericidal concentrations were conducted against *Escherichia coli* at different concentrations of honey samples ranging from 60% to 1%. Our results revealed that both MIC and MBC were the same; SH was 50%, and MH was 30%. A previous study conducted against a clinical isolate of *E. coli* and SH showed a different result in which the MIC was 10 %, while MBC was 40% [20]. The MIC result obtained from [3] of SH against *E. coli* was 40%, whereas MBC was 60%. Another study conducted by Tan et al. [22], who compared MIC between MH and Malaysian Tualang honey against bacterial wounds and enteric microbial by spectrophotometer and visual inspection, found that the MIC for MH ranges between 8.75% to 20%, whereas the MIC for Malaysian honey ranges between 8.75% to 25% (*w/v*). In this study, MH exhibited more effectiveness (30%) than SH (50%). As a result, determining the exact bactericidal effect of MH remains unknown [5,6,19,23]. However, methylglyoxal (MGO) was a primary bactericidal agent in MH. Even after being neutralized, it maintains its activities due to other unknown constituents in MH, which may synergistically exert these antibacterial activities [24]. Generally, honey’s antibacterial effect has been related to the presence of MGO in MH and hydrogen peroxide in SH. Other factors that contribute to the antibacterial activities of honey include low pH, osmotic pressure, bee defensin-1 (an antibacterial peptide derived from bees), low protein content, and hyper-osmolality impact. The pH of honey ranges between 3.5 and 5.5, while most bacteria grow best at neutral pH ranging from 6.5 to 7.5 [8]. The high sugar content of honey results in high osmotic pressure and causes water to flow out from the bacterial cells through osmosis. As a consequence of the dehydration and high osmotic pressure, the bacteria cells start to shrink and cannot grow in the hypertonic sugar solution [25]. Phytochemical diversity, phenolic and flavonoid contents, and the high carbon-to-nitrogen ratio are other contributing factors to the antimicrobial activities of honey [26].

The antibacterial mechanisms of action of honey samples against *E. coli* were carried out using several assays, including potassium ions and protein leakages from the bacterial cell membrane, bacterial protein inhibition, and DNA damage. The quantities of potassium ions and proteins leaked from *E. coli* increased over time. The bacterial cytoplasmic membrane acts as a selective barrier to small ions such as Ca2+, Na+, and K+, prohibiting their input and outflow from the cells. The control of cellular membrane permeability is a critical regulator for various cellular functions, including solute transport, energy transduction, cell metabolism maintenance, and other functions [27,28]. The cytoplasmic membrane disruption caused by antibacterial agents could allow cations or other constituents to be readily transferred out from the cell, for instance, amino acids, K+, proteins, nucleic acids, and inorganic phosphates, resulting in cell lysis and death. The potassium ion is important in maintaining a consistent internal pH and membrane potential [28,29,30]. The permeability of the cytoplasmic membrane of *E. coli* was considerably impaired by the addition of honey into the bacterial suspension, particularly both concentrations of SH, as indicated by the remarkable increase in potassium and protein leakage into the bacterial suspension.

Phytochemical compounds such as flavonoids and phenolic acids in honey are implicated in antibacterial characteristics in defense systems. The possible bioactive phytochemical compounds found in both honey types that may exhibit antibacterial properties according to previous literature include MGO in MH [31], hydrogen peroxide in SH [32], phenolic acids and flavonoids such as p-coumaric acid, gallic acid, ferulic acid, myricetin, and rosmarinic acid [1,31,33,34]. Previous studies carried out against various pathogenic bacterial strains involving *E. coli* (ATCC 25922) revealed that p-coumaric acid suppressed bacterial growth while increasing the permeability of the outer plasma membranes, causing the loss of the barrier function [33,35,36]. Another study reported by Borges et al. [37] tested the role of the mechanism of action of ferulic and gallic acids against *E. coli* and other pathogenic bacteria and found that both of them caused irreversible damages in membrane characteristics such as extra and intracellular permeability, physicochemical features and charge loss by hydrophobicity alteration, the emergence of localized rupture or increased pore formation and decline of negative surface charge in the cell membrane [31,33,36]. Another published study reported that ferulic acid has antibacterial properties against *Cronobacter sakazakii* and was thought to act on the cell membrane through a substantial change in intracellular ATP concentrations, a decline in intracellular pH, cell membrane hyperpolarization, a depreciation in bacterial membrane integrity, and morphological alterations [38]. Another study by Halawani [39] treated *E. coli* with diluted Shaoka honey and observed under electron microscopy that bacterial cells had altered cell morphology involving shrinkage in bacterial size resulting in reduction and leaking of intracellular content as well as cell-wall breakdown, which led to cell death. Other researchers have indicated that the antibacterial mechanisms of flavonoids are expected to have other actions, such as suppression of nucleic acid synthesis, modulation of cytoplasmic membrane function, deformation of membrane permeability, and pathogenicity attenuation [40,41]. Protein inhibition of *E. coli* demonstrated that both honey samples caused decreases in protein synthesis in a time-dependent manner, particularly in MH-treated bacteria. Rosmarinic acid could play a role in the inhibition of bacterial protein synthesis [36,42]. MH can alter bacterial form and size depending on the bacterial strains, impacting the septal ring related to cell division. Henriques et al. [43] studied the impact of MH on *S. aureus* culture using transmission electron microscopy and reported that tested bacterial strains had more septal cells than those treated with artificial honey prepared from sugars and water, which resembled the composition of natural honey. A phase-contrast imaging investigation showed that treating *Bacillus subtilis* and *S. aureus* with a sub-lethal dose of MH (4%) resulted in the appearance of smaller cells with condensed chromosomes [44]. P-coumaric acid can attach to the phosphate anion in the double helix of DNA, potentially affecting replication, transcription, and expression. It has two bactericidal mechanisms: it disrupts bacterial cell membranes and binds to bacterial genomic DNA to block cellular activities, resulting in cell death [35]. Myricetin is another natural flavonoid substance that has been shown to disrupt a variety of RNA polymerases, DNA polymerases, telomerase and reverse transcriptases, helicases, and kinases. Myricetin inhibits *E. coli* DNA B helicase at physiological ATP concentrations with an inhibitory concentration at 50% maximum (IC_50_) of 11.3 ± 1.6 μM [33,45]. To sum up, the findings of our study confirmed that the mechanisms of action of SH and MH at the concentrations of 1x and 2x MIC against *E. coli* (ATCC 25922) experienced intense alterations in the bacterial cells, elucidating the effect of honey samples on their cell membrane permeability, integrity, protein, and nucleic acids dysfunction on the bacterial cells, ultimately causing cellular degradation and death.

One conclusion is that the efficacy of two types of honey against *E. coli* was contingent on the concentration administered. The differences in concentrations of MGO, H_2_O_2_ and other bioactive constituents among honey types have resulted in different bactericidal activities. These are key considerations for optimizing honey for wound infection management since sub-lethal honey levels may have unexpected consequences [44]. Honey that does not contain considerable amounts of MGO or H_2_O_2_, such as clover honey, may be able to suppress some bacteria but does not exert a broad-spectrum activity and is thus not advised for infected wounds where numerous species may be present [46]. Due to the antibacterial potency of MH, MGO has been utilized as a preservative in natural cosmetics. The replacement of synthetic preservatives reduces microbial contamination and provides a safe and natural solution [46].

## 4. Materials and Methods

### 4.1. Honey Samples

Two honey samples were used, namely Yemeni Sidr honey (SH), which was obtained from the local Yemeni market in Malaysia (Arab house-Serdang), and Manuka honey (MH) (MGO 400+), which was purchased from a pharmacy in Malaysia. The honey samples were kept away from sunlight at room temperature.

### 4.2. Bacterial Strain

*Escherichia coli* (ATCC 25922), a pathogenic bacterial strain associated with wound infection, was used as a tested microorganism in this study; this strain belongs to Gram-negative bacteria. The isolated bacterial strain was sub-cultured on tryptic soy agar (Sigma-Aldrich, St. Louis, MO, USA), incubated aerobically at 37 °C for 24 h and then maintained in the laboratory at 4 °C [47,48]. 

### 4.3. Determination of Antibacterial Susceptibility Assay

The antimicrobial susceptibility testing of honey samples against *E. coli* was determined by the disc diffusion method described by Bauer et al. at the honey concentration of 700 mg/disc [49]. Briefly, bacterial colonies were isolated from pure culture and inoculated in 5 mL of tryptic soy broth (Merck, Darmstadt, Germany), then incubated at 37 °C for 2–5 h [49]. The optical density (O.D) was measured spectrophotometrically and adjusted between 0.08 and 0.1 at 600 nm, which was approximately equal to 10^8^ CFU/mL (corresponding to 0.5 McFarland standard) [48,50]. Subsequently, bacterial suspension was spread evenly on an agar plate by a sterile cotton swab. Sterile 6.0 mm diameter blank discs (HiMedia, Mumbai, India) were impregnated with 50 µL of the honey concentration and vehicle as a negative control separately, then placed on the surface of the inoculated plate using sterile forceps. Gentamicin 10 µg/disc (Himedia, Mumbai, India) was used as a positive reference control. The plates were incubated aerobically at 37 °C for 24 h. The inhibition zone diameter was measured in mm, and the average was calculated. The experiment was repeated in triplicates for each isolate.

### 4.4. Determination of Inhibitory Concentration (MIC) and Minimum Bactericidal Concentration (MBC)

According to the Clinical and Laboratory Standards Institute, the MIC and MBC were determined using the broth macrodilution method [51]. The minimum concentration of the tested honey that completely inhibits microbial growth was estimated as the MIC value. In contrast, the minimum concentration showing the complete absence of the bacterial colonies’ growth on the plate was considered the MBC value. Sterile Eppendorf tubes with the different final concentrations of honey constituting 60%, 50%, 40%, 30%, 20%, 10%, 5%, and 1% (*v*/*v*) were made using tryptic soy broth (Merck, Darmstadt, Germany); this was done by dissolving the respective volumes of 600, 500, 400, 300, 200, 100, 50, and 10 µL of each honey into the corresponding volume of sterile broth medium to give a 1 mL of honey solution preparation. Then, 100 µL of bacterial suspension (1.5 × 10^8^ CFU/tube) was dispensed into the tubes. The negative control tube contained the broth medium (no bacteria), while the positive control tube contained the broth medium with bacteria. The tubes were incubated at 37 °C for 24 h. The MIC was observed visually for the presence or absence of bacterial growth turbidity compared with the negative control. All tubes showing no visible growth were inoculated onto sterile nutrient agar plates by streak plate and incubated further at 37 °C overnight. On the second day, the lowest concentration showing no signs of the bacterial colony on agar indicated the MBC value.

### 4.5. Determination of Potassium (K^+^) Ions Leakage from the Cell Membrane

The leakage of free potassium (K+) ions from *E. coli* was investigated to detect the breakdown of the permeability of bacterial cell membrane as described by Patra and Baek [28]. Briefly, after determining the MIC value of each honey, 100 μL of bacterial suspension (1.5 × 10^8^ CFU/tube) was added to 1 mL of SH and MH at 1x and 2x MIC concentrations with sterile peptone water (Merck, Darmstadt, Germany) and subsequently incubated at 37 °C for 4 h. Through this time, the extracellular K+ concentration was determined each hour by a photometric procedure using the kalium/potassium kit (Quantofix, Macherey-Nagel GmbH & Co. KG, Duren, Germany). The bacterial culture tube without honey was used as a negative control. The results were expressed as extracellular free potassium ions in mg/L (in the bacterial suspension) at each time interval of incubation.

### 4.6. Determination of Bacterial Protein Leakage from the Cell Membrane

The leakage of protein from *E. coli* through the cell membrane was conducted as described by Abegunde et al. [52] with a slight modification. In brief, approximately 18 h of the tryptic soy broth bacterial suspension was washed three times in 0.9% *w/v* normal saline. Then, the bacterial suspension was adjusted to be nearly 1.5 × 10^8^ cells/mL by 0.5 McFarland standard. Samples of 1 mL of 1x, 2x MIC, and negative control (bacterial suspension) were added into the Eppendorf tube; then, 100 µL of bacterial suspension were added and subsequently incubated at 37 °C at 0, 30, 60, 120, and 180 min. After that, each suspension was centrifuged at 7000 rpm, and the supernatant was collected for the protein leakage measurement using the Bradford assay (Cayman protein determination kit, item No:704002, Ellsworth Rd., Ann Arbor, MI, USA). A 40 µL of Bradford reagent was added to 160 µL of samples (20 µL of supernatant plus 140 µL of sterile distilled water) to reach up to 2 mL of total volume. The absorbance was read at 595 nm, and bovine serum albumin (BSA) was used as a standard to obtain the calibration curve.

### 4.7. Determination of Inhibition of Bacterial Protein

The protein content of treated and untreated *E. coli* was performed as described by Shu et al. [42] with minor modifications to evaluate the inhibitory effects of honey samples on the protein of *E. coli*. Briefly, overnight the tryptic soy broth bacterial suspension was washed three times in 0.9% *w*/*v* normal saline and then adjusted to nearly 10^8^ cells/mL. After that, 800 µL of honey samples at the 1x, 2x MIC, and negative control (bacterial suspension) were prepared in sterile glass tubes. Subsequently, 200 µL of bacterial suspension were added, mixed well, and incubated at 37 °C at various time intervals for 24 h. During this time, the samples were disrupted by ultrasonic sonication (working for 3 s at 5 s intervals for 5 min in an ice bath) and subsequently centrifuged at 7000 rpm to remove cell debris, and then the supernatant was collected to detect protein content using the Bradford method (Cayman protein determination kit, item No:704002, Ellsworth Rd., Ann Arbor, MI, USA). About 160 µL of samples (20 µL of supernatant plus 140 µL of sterile distilled water) were dispensed in a 96-well plate; then, 40 µL of Bradford reagent were added to reach up to 2 mL of total volume. The absorbance was read at 595 nm after 5 min. Bovine serum albumin (BSA) was used to obtain the calibration curve.

### 4.8. Evaluation of Bacterial DNA Damage

The biochemical induction assay (BIA) is one of the sensitive methods that directly or indirectly measure the DNA damage in which activation of the LacZ gene (located in a bacterial nucleic acid) encodes β-galactosidase production. When the bacterial culture is exposed to a DNA-damaging agent, the LacZ gene is transcribed and fused. Consequently, the enzyme β-galactosidase is dispensed into the culture, which is identified by a suitable chromogenic reagent substrate reacting with the enzyme resulting in the production of color around the test compound, which is used as a DNA-damaging induction in the colorimetric broth micro-dilution method [53]. This assay was carried out according to the method described by Hamed El-Batanony [54] with slight modifications. In short, a sterile cotton swab of *E. coli* suspension (10^8^ CFU/mL) was evenly dispensed on agar plates and incubated at 37 °C for 48 h. The plates were spotted with 1x, 2x MIC, 10 µg/mL ciprofloxacin (positive control), and PBS (negative control), then incubated at 37 °C for 3 h. After incubation, the plates were overlaid with a methanolic solution of 6-Bromo-2-naphthyl B-D-glucopyranoside (8 mg/mL) (Sigma-Aldrich, Louis, MO, USA). Subsequently, the plates were incubated for 2 to 4 h at 37 °C and then covered with an aqueous solution of Fast Blue B Salt (5 mg/mL); after that, they were left for 10 min. A bacterial smear was prepared on a clean glass slide, then stained with a Gram stain (Sigma-Aldrich, St. Louis, MO, USA) following the Gram staining kit. β-galactosidase activity (DNA damage) was estimated by an intense dark purple dye under an inverted microscope at an oil immersion lens.

### 4.9. Statistical Analysis

Statistical analysis was performed using SPSS (Statistical Package for the Social Sciences) version 26.0 for Windows. Data analyses were done for at least three independent experiments and shown as the mean ± SEM of the indicated replicates.

## 5. Conclusions

In conclusion, this study showed that SH and MH had antibacterial properties against *Escherichia coli.* Furthermore, the underlying mechanisms of action of both honey types against *E. coli* occur mainly through interfering with the bacterial membrane permeability, increasing potassium ion and protein leakage, inhibiting proteins and inducing bacterial DNA damage.

## Data Availability

Not applicable.

## References

[1] Alvarez-Suarez J.M., Gasparrini M., Forbes-Hernandez T.Y., Mazzoni L., Giampieri F. (2014). The Composition and Biological Activity of Honey: A Focus on Manuka Honey. Foods.

[2] Vallianou N.G., Gounari P., Skourtis A., Panagos J., Kazazis C. (2014). Honey and its anti-inflammatory, anti-bacterial and anti-oxidant properties. Gen. Med..

[3] Ghramh H.A., Khan K.A., Alshehri A.M.A. (2019). Antibacterial potential of some Saudi honeys from Asir region against selected pathogenic bacteria. Saudi J. Biol. Sci..

[4] Snowdon J.A., Cliver D.O. (1996). Microorganisms in honey. Int. J. Food Microbiol..

[5] Szweda P. (2017). Antimicrobial activity of honey. Honey Anal..

[6] Adams C.J., Boult C.H., Deadman B.J., Farr J.M., Grainger M.N., Manley-Harris M., Snow M.J. (2008). Isolation by HPLC and characterisation of the bioactive fraction of New Zealand manuka (*Leptospermum scoparium*) honey. Carbohydr. Res..

[7] Cianciosi D., Forbes-Hernández T.Y., Afrin S., Gasparrini M., Reboredo-Rodriguez P., Manna P.P., Zhang J., Bravo Lamas L., Martínez Flórez S., Agudo Toyos P. (2018). Phenolic compounds in honey and their associated health benefits: A review. Molecules.

[8] Pauliuc D., Dranca F., Oroian M. (2020). Antioxidant Activity, Total Phenolic Content, Individual Phenolics and Physicochemical Parameters Suitability for Romanian Honey Authentication. Foods.

[9] Molan P.C. (1999). The role of honey in the management of wounds. J. Wound Care.

[10] Kumar M.S., Sripriya R., Raghavan H.V., Sehgal P.K. (2006). Wound healing potential of Cassia fistula on infected albino rat model. J. Surg. Res..

[11] James O., Victoria I.A. (2010). Excision and incision wound healing potential of *Saba florida* (Benth) leaf extract in Rattus novergicus. Inter. J. Pharm. Biomed. Res..

[12] Sankar R., Baskaran A., Shivashangari K.S., Ravikumar V. (2015). Inhibition of pathogenic bacterial growth on excision wound by green synthesized copper oxide nanoparticles leads to accelerated wound healing activity in Wistar Albino rats. J. Mater. Sci. Mater. Med..

[13] Girma A., Seo W., She R.C. (2019). Antibacterial activity of varying UMF-graded Manuka honeys. PLoS ONE.

[14] Alsaud N., Shahbaz K., Farid M. (2021). Application of deep eutectic solvents in the extraction of polyphenolic antioxidants from New Zealand Manuka leaves (*Leptospermum scoparium*): Optimization and antioxidant activity. J. Mol. Liq..

[15] Al Refaey H.R., Newairy A.-S.A., Wahby M.M., Albanese C., Elkewedi M., Choudhry M.U., Sultan A.S. (2021). Manuka honey enhanced sensitivity of HepG2, hepatocellular carcinoma cells, for Doxorubicin and induced apoptosis through inhibition of Wnt/β-catenin and ERK1/2. Biol. Res..

[16] Obossou E.K., Shikamoto Y., Hoshino Y., Kohno H., Ishibasi Y., Kozasa T., Taguchi M., Sakakibara I., Tonooka K., Shinozuka T. (2022). Effect of manuka honey on human immunodeficiency virus type 1 reverse transcriptase activity. Nat. Prod. Res..

[17] Cianciosi D., Regolo L., Battino M., Giampieri F. (2021). Effect of manuka honey on 5-fluorouracil chemosensitivity in colonspheres enriched with cancer stem (-like) cells. Biomed. Sci. Eng..

[18] Abd-El Aal A.M., El-Hadidy M.R., El-Mashad N.B., El-Sebaie A.H. (2007). Antimicrobial effect of bee honey in comparison to antibiotics on organisms isolated from infected burns. Ann. Burn. Fire Disasters.

[19] Mundo M.A., Padilla-Zakour O.I., Worobo R.W. (2004). Growth inhibition of foodborne pathogens and food spoilage organisms by select raw honeys. Int. J. Food Microbiol..

[20] Saeed M.A., Jayashankar M. (2020). Evaluation of Antibacterial Activity of some Indian and Yemeni Honey against Few Bacterial Isolates from Human Patients. Egypt. J. Microbiol..

[21] Luka B.R. (2021). Comparison of the antibacterial effect of manuka honey and domestic acacia honey. AGRORES.

[22] Tan H.T., Rahman R.A., Gan S.H., Halim A.S., Hassan S.A., Sulaiman S.A., Kirnpal-Kaur B. (2009). The antibacterial properties of Malaysian tualang honey against wound and enteric microorganisms in comparison to manuka honey. BMC Complement. Altern. Med..

[23] Molan P.C. (1992). The antibacterial activity of honey: 1. The nature of the antibacterial activity. Bee World.

[24] Kwakman P.H., Te Velde A.A., De Boer L., Vandenbroucke-Grauls C.M., Zaat S.A. (2011). Two major medicinal honeys have different mechanisms of bactericidal activity. PLoS ONE.

[25] Molan P., Rhodes T. (2015). Honey: A Biologic Wound Dressing. Wounds A Compend. Clin. Res. Pract..

[26] Johnston M., McBride M., Dahiya D., Owusu-Apenten R., Nigam P.S. (2018). Antibacterial activity of Manuka honey and its components: An overview. AIMS Microbiol..

[27] Cox S., Gustafson J., Mann C., Markham J., Liew Y.C., Hartland R., Bell H.C., Warmington J., Wyllie S.G. (1998). Tea tree oil causes K+ leakage and inhibits respiration in Escherichia coli. Lett. Appl. Microbiol..

[28] Patra J.K., Baek K.H. (2016). Antibacterial Activity and Action Mechanism of the Essential Oil from Enteromorpha linza L. against Foodborne Pathogenic Bacteria. Molecules.

[29] Gründling A. (2013). Potassium uptake systems in Staphylococcus aureus: New stories about ancient systems. mBio.

[30] Lambert P.A., Hammond S.M. (1973). Potassium fluxes, first indications of membrane damage in micro-organisms. Biochem. Biophys. Res. Commun..

[31] Oelschlaegel S., Gruner M., Wang P.N., Boettcher A., Koelling-Speer I., Speer K. (2012). Classification and characterization of manuka honeys based on phenolic compounds and methylglyoxal. J. Agric. Food Chem..

[32] Noori A., Al Ghamdi A., Ansari M.J., Al-Attal Y., Al-Mubarak A., Salom K. (2013). Differences in composition of honey samples and their impact on the antimicrobial activities against drug multiresistant bacteria and pathogenic fungi. Arch. Med. Res..

[33] Olas B. (2020). Honey and its phenolic compounds as an effective natural medicine for cardiovascular diseases in humans?. Nutrients.

[34] Badjah Hadj Ahmed A., Obbed M.S., Wabaidur S.M., AlOthman Z.A., Al-Shaalan N.H. (2014). High-performance liquid chromatography analysis of phenolic acid, flavonoid, and phenol contents in various natural Yemeni honeys using multi-walled carbon nanotubes as a solid-phase extraction adsorbent. J. Agric. Food Chem..

[35] Lou Z., Wang H., Rao S., Sun J., Ma C., Li J. (2012). p-Coumaric acid kills bacteria through dual damage mechanisms. Food Control.

[36] Anand S., Deighton M., Livanos G., Morrison P.D., Pang E.C.K., Mantri N. (2019). Antimicrobial Activity of Agastache Honey and Characterization of Its Bioactive Compounds in Comparison with Important Commercial Honeys. Front. Microbiol..

[37] Borges A., Ferreira C., Saavedra M.J., Simoes M. (2013). Antibacterial activity and mode of action of ferulic and gallic acids against pathogenic bacteria. Microb. Drug Resist..

[38] Shi C., Zhang X., Sun Y., Yang M., Song K., Zheng Z., Chen Y., Liu X., Jia Z., Dong R. (2016). Antimicrobial Activity of Ferulic Acid Against Cronobacter sakazakii and Possible Mechanism of Action. Foodborne Pathog. Dis..

[39] Halawani E.M. (2021). Potential effects of Saudi Shaoka (*Fagonia bruguieri*) honey against multi-drug-resistant bacteria and cancer cells in comparison to Manuka honey. Saudi J. Biol. Sci..

[40] Wen C.T.P., Hussein S.Z., Abdullah S., Karim N.A., Makpol S., Yusof Y.A.M. (2012). Gelam and nenas honeys inhibit proliferation of HT 29 colon cancer cells by inducing DNA damage and apoptosis while suppressing inflammation. Asian Pac. J. Cancer Prev..

[41] Jibril F.I., Hilmi A.B.M., Manivannan L. (2019). Isolation and characterization of polyphenols in natural honey for the treatment of human diseases. Bull. Natl. Res. Cent..

[42] Shu H., Chen H., Wang X., Hu Y., Yun Y., Zhong Q., Chen W., Chen W. (2019). Antimicrobial Activity and Proposed Action Mechanism of 3-Carene against *Brochothrix thermosphacta* and *Pseudomonas fluorescens*. Molecules.

[43] Henriques A.F., Jenkins R.E., Burton N.F., Cooper R.A. (2010). The intracellular effects of manuka honey on Staphylococcus aureus. Eur. J. Clin. Microbiol. Infect. Dis. Off. Publ. Eur. Soc. Clin. Microbiol..

[44] Lu J., Carter D.A., Turnbull L., Rosendale D., Hedderley D., Stephens J., Gannabathula S., Steinhorn G., Schlothauer R.C., Whitchurch C.B. (2013). The effect of New Zealand kanuka, manuka and clover honeys on bacterial growth dynamics and cellular morphology varies according to the species. PLoS ONE.

[45] Griep M.A., Blood S., Larson M.A., Koepsell S.A., Hinrichs S.H. (2007). Myricetin inhibits Escherichia coli DnaB helicase but not primase. Bioorganic Med. Chem..

[46] Juliano C., Magrini G.A. (2019). Methylglyoxal, the major antibacterial factor in manuka honey: An alternative to preserve natural cosmetics?. Cosmetics.

[47] Abd Mohammed Ali S. (2018). Comparison of the effect of natural and commercial honey on the growth and Antibiotic sensitivity of *Escherichia coli* and *Pseudomonas aeruginosa*. Ann. Agric. Sci. Moshtohor..

[48] Clinical and Laboratory Standards Institute (2009). Performance Standards for Antimicrobial Susceptibility Testing of Anaerobic Bacteria: Informational Supplement.

[49] Bauer A.W., Kirby W.M., Sherris J.C., Turck M. (1966). Antibiotic susceptibility testing by a standardized single disc method. Am. J. Clin. Pathol..

[50] Humphries R.M., Ambler J., Mitchell S.L., Castanheira M., Dingle T., Hindler J.A., Koeth L., Sei K., Kraft C.S. (2018). Standardization Working Group of the Subcommittee on Antimicrobial Susceptibility, T. CLSI Methods Development and Standardization Working Group Best Practices for Evaluation of Antimicrobial Susceptibility Tests. J. Clin. Microbiol..

[51] Ruangpan L., Tendencia E.A. (2004). Laboratory Manual of Standardized Methods for Antimicrobial Sensitivity Tests for Bacteria Isolated from Aquatic Animals and Environment.

[52] Abegunde M., Akinpelu D., Omololu-Aso J., Otusanya O., Akinlolu J. (2018). Determination of antimicrobial, antioxidant and phytochemical properties of Cocos nucifera linn Endocarp extract on bacteria associated with human infection. J. Pharm. Microbiol..

[53] Singh M.P., Arias D.A., Greenstein M. (2005). Chemiluminometric biochemical induction assay (CBIA) for the detection of DNA-damaging agents. J. Microbiol. Methods.

[54] Hamed El-Batanony N. (2017). Antimicrobial activities and mode of action of the selected novel thienopyrimidines derivatives 2-[2-(diphenylmethylene) hydrazino]-5-isopropyl-3-methylthieno [2, 3-d] pyrimidin-4-one. Period. Biol..

